# Effects of Different Doses of Glucosamine Hydrochloride on Cartilage Tissue and Levels of Joint Injury Markers in Knee Osteoarthritis

**DOI:** 10.1111/jcmm.70579

**Published:** 2025-05-19

**Authors:** Xichun Wang, Bin Hu, Yi Cheng, Wenjie Chen, Muzi Liu

**Affiliations:** ^1^ Department of Orthopedics, Jiujiang City Key Laboratory of Cell Therapy Jiujiang No. 1 People's Hospital Jiujiang China; ^2^ Department of Oncology Jingshan People's Hospital Jingmen China

**Keywords:** cartilage tissue, different doses, glucosamine hydrochloride, joint injury, knee osteoarthritis, markers

## Abstract

This study analysed the effects of different doses of glucosamine hydrochloride (GS‐HCl) on cartilage tissue and the levels of joint injury markers in knee osteoarthritis (KOA). The Sham group, KOA group, low‐dose GS‐HCl group and high‐dose GS‐HCl group were established, with six mice in each group. The levels of joint injury markers (COMP, CS846 and CTX‐II), inflammatory cytokines (IL‐6, TNF‐α and iNOS), oxidative stress indicators (MDA and SOD) and matrix remodelling proteins (MMP‐3 and TIMP‐1) were analysed. The degeneration of knee femoral condyles, histopathological changes and tissue apoptosis rate of the articular cartilage was also assessed. Mice in the KOA group displayed elevated COMP, CS846, CTX‐II, IL‐6, TNF‐α, iNOS and MDA contents, reduced SOD activity, an irregular articular cartilage surface, a serious cartilage defect, a disordered articular cartilage surface in the defect, disappeared cartilage cells, obvious synovial cell proliferation and visible inflammatory cell infiltration. In the tissue, apoptosis rate and MMP‐3 and TIMP‐1 protein expression increased. Different doses of GS‐HCl treatment could reduce COMP, CS846, CTX‐II, IL‐6, TNF‐α, iNOS and MDA contents, apoptosis rate and MMP‐3 and TIMP‐1 protein expression, increase SOD activity and improve histopathological conditions in KOA mice. The improvement effects in each indicator in the high dose–GS‐HCl group were more significant than those in the low dose–GS‐HCl group. The intragastric administration of the GS‐HCl group partially prevents the degeneration of articular cartilage in KOA mice. The mechanism may be to reduce inflammatory factors and oxidative stress indicator expression and matrix degradation, thereby delaying osteoarthritis progression.

## 1 Introduction

Knee osteoarthritis (KOA) refers to a heterogeneous pathological process featured with a complex pathophysiology [1]. It generally manifests as joint pain that worsens with activity and improves during periods of rest. It is characterised by short‐lived, self‐resolving morning stiffness and does not cause general body‐wide symptoms [2]. Among all joint diseases, the incidence of KOA ranks at the forefront, and it is likely to increase in the coming decades [3]. KOA is a primary contributor to disability, incurring significant healthcare expenses, and there remains a need for effective non‐surgical treatment approaches [4].

Individuals in the early or middle stages of KOA typically undergo conservative treatment. Injections of compound osteopeptide are frequently administered, as they comprise cytokines, amino acids, bone polypeptides and other constituents that can promote bone growth and modulate bone metabolism [5]. In addition to these treatment methods, glucosamine, a natural component in cartilage, has also been proven beneficial for joint health and can even reduce mortality rates [6]. Glucosamine is a crucial component for delaying cartilage decomposition and repairing damaged cartilage, exerting a protective effect on articular cartilage and promoting knee joint function [5]. Glucosamine hydrochloride (GS‐HCl), as one of the most basic and important derivatives of chitin, can be obtained by hydrolysing chitin with concentrated hydrochloric acid [7]. It has been reported that GS‐HCl is a natural compound found in various human tissues and participates in the construction of cell membranes and human tissues. Moreover, GS‐HCl also plays a significant role in the repair of osteogenic injuries [8]. It is also reported that GS‐HCl acts as an amino monosaccharide in the treatment of KOA [9]. A previous study has demonstrated that GS‐HCl is capable of suppressing cell development and matrix synthesis [10]. GS‐HCl utilisation can improve knee pain, reduce arthritis symptoms, promote knee function and reduce the expression levels of inflammatory cytokines in KOA patients [11]. Other studies have shown that the application of GS‐HCl can lead to efficacious anti‐arthritic effects [12]. Furthermore, in adjuvant arthritis, the benefits of oral glucosamine are dose‐dependent [13]. Considering the above research, we realised that the effects of different doses of GS‐HCl in KOA were rarely discussed and the protective role of different doses of GS‐HCl in KOA was worthy of deep analysis. Therefore, this study focused on analysing the protective impact of different doses of GS‐HCl on cartilage tissue of KOA and its effects on the levels of joint injury markers.

## 2 Materials and Methods

### 2.1 Ethics Statement

The experimental animals were utilised for medical research and all operations were conducted under the discussion and ratification of Jiujiang City Key Laboratory of Cell Therapy, Jiujiang No. 1 People's Hospital.

### 2.2 KOA Mouse Models

Twenty‐four 10‐week‐old male specific pathogen‐free (SPF)‐grade C57BL/6 mice weighted 20–25 g were provided by Hunan Xintu Medical Diagnostic Technology Co. Ltd. (Certification number: SYXK (Xiang) 2023–0013). All animals were kept in an SPF‐grade animal house at 27°C, with 50% humidity and free access to water and food, and at a 12 h/12 h light–dark cycle. All animals were acclimatised for 1 week, and the experiments were started after no abnormalities or diseases were observed. The mice were separated into four groups including Sham group, KOA group, low‐dose GS‐HCl group and high‐dose GS‐HCl group by utilising the random number table method, with 6 mice in each group. KOA mouse models were established by anterior cruciate ligament transection (ACLT): The experimental mice were anaesthetised by intraperitoneal injection of 5% chloride hydrate and then put in a supine position. Next, a longitudinal incision of approximately 7 mm was made in the medial aspect of the knee joint, and then subcutaneous tissues were peeled off layer by layer. The anterior cruciate ligament was cut off with an ophthalmic scalpel under an in‐body microscope, the bleeding was stopped, and the surgical incision was closed. Mice in the Sham group only had the right knee joint cavity exposed [14]. Starting on the first day after modelling, mice in the low‐dose GS‐HCl and high‐dose GS‐HCl groups were gavaged daily with the corresponding dose of GS‐HCl for 8 weeks, and those in the Sham and KOA groups were gavaged with saline for 8 weeks. The drug intake in humans and mice was converted according to MeehRubner's formula A = K × W^2/3^ [A was a surface area (m^2^); W was body weight (kg)] [15, 16], and the specific daily gavage doses were 41.5 mg/kg for the low‐dose GS‐HCl group and 137.5 mg/kg for the high‐dose GS‐HCl group.

### 2.3 Enzyme‐Linked Immunosorbent Assay (ELISA)

The levels of serum cartilage oligomeric matrix protein (COMP), chondroitin sulfate 846 (CS846), C‐terminal telopeptide of type II collagen (CTX‐II), interleukin‐6 (IL‐6), tumour necrosis factor (TNF)‐α and inducible nitric oxide synthase (iNOS) were assessed by ELISA. Serum specimens were taken, centrifuged at 5000× g for 10 min and subjected to ELISA. The absorbance (OD) values of each well were measured at 450 nm by utilising a microplate reader. The ELISA kits for COMP (CSB‐EL005778MO), CTX‐II (CSB‐E16211m), IL‐6 (CSB‐E04639m), TNF‐α (CSB‐E04741m) and iNOS (CSB‐E08326m) were all obtained from Wuhan Huamei BIOTECH Co. Ltd. (Wuhan, Hubei, China), and the CS846 ELISA kit was purchased from IBEX Pharmaceuticals Inc. (Canada) [17].

### 2.4 Evaluation of OA severity

The severity of OA was assessed adopting H&E and Safranin‐O staining, as well as modified Mankin scoring. Knee tissues were fixed in 4% paraformaldehyde for 24 h, decalcified in 10% EDTA solution for 4 weeks, dehydrated in conventional gradient ethanol, impregnated and embedded in paraffin and sectioned in 4‐μm‐thick sections. The HE staining kit (Beyotime, Shanghai, China) was used for staining, and three to five fields of view were randomly taken from each section to observe the histological and morphological characteristics of cartilage and chondrocytes under a 400× light microscope. The changes were scored according to the modified Mankin scoring criteria [18], and higher scores indicated a higher lesion degree of osteoarthritis: 0–1 point for normal articular cartilage, 2–5 points for mild lesions, 6–9 points for moderate lesions and 10–14 points for severe lesions. The scoring was conducted blindly by two independent researchers. If the score difference exceeded 2 points, a third researcher re‐evaluated. The consistency of the scores from three fields of view was confirmed by intraclass correlation coefficient (ICC) analysis (ICC > 0.9) to ensure the reliability of the results. The average of the 3 scores was taken as the final score [19].

### 2.5 Terminal Deoxynucleotidyl Transferase (TdT) dUTP Nick‐End Labeling (TUNEL) Assay

Paraffin sections were sliced and put into water, covered with drops of proteinase K working solution, and then the slices were incubated at 37°C for 30 min, and rinsed three times in PBS for 5 min each time. Next, the slices were slightly dried and covered with drops of membrane‐breaking working solution, and the above operation was repeated. Cell apoptosis was identified by implementing the TUNEL kit (Wuhan Procell Life Science & Technology Co. Ltd., Wuhan, China). Four section areas were randomly examined under a fluorescence microscope, and the results were expressed as the percentage of TUNEL‐positive cells in the selected area (TUNEL‐positive cells/total number of nuclei × 100%) [20].

### 2.6 Determination of Oxidative Stress Indicators

The serum of each group was collected and centrifuged at 2000 g for 15 min. The supernatant was measured by superoxide dismutase (SOD) kit (WST‐1 method, A001‐3‐2) and malondialdehyde (MDA) kit (TBA method, A003‐1‐2), respectively. The kits were purchased from Nanjing Jiancheng Biological Engineering Research Institute (Nanjing, Jiangsu, China). The absorbance of SOD (at 450 nm) and MDA (at 532 nm) was measured using a microplate reader (Multiskan GO, Thermo Fisher) [21].

### 2.7 Western Blot Assay

Articular cartilage tissues of each group were weighed and crushed with bone forceps, then quickly crushed in a mortar with the addition of 5 mL of liquid nitrogen, added with 1 mL of pre‐cooled Lysis Buffer (10 μL of phosphatase inhibitor, 1 μL of protease inhibitor and 5 μL of 100 mM phenylmethylsulfonyl fluoride (PMSF) were supplemented and mixed well) and centrifuged in a centrifuge at 12,000 r/min for 30 min; the supernatant was taken to measure protein concentration. Protein samples were separated by 10% sodium dodecyl sulfate polyacrylamide gel electrophoresis (SDS‐PAGE) and transferred to polyvinylidene fluoride (PVDF) membranes. The membranes were rinsed with Tris buffered saline with Tween (TBST) buffer, blocked in 5% skimmed milk powder solution at ambient temperature for 1 h, supplemented with diluted primary antibodies matrix metalloproteinase‐3 (MMP‐3) (Abcam, Cambridge, MA, UK, ab52915, 1:1000), tissue inhibitor of metalloproteinase‐1 (TIMP‐1) (Abcam, ab179580, 1:1000) or anti‐glyceraldehyde‐3‐phosphate dehydrogenase (GAPDH) (Abcam, 1: 10000, ab128915) and incubated at 4°C overnight. The next day, after rinsing with TBST buffer, the membranes were cultivated with horseradish peroxidase (HRP)‐labelled secondary antibody (Abcam, ab205718) at ambient temperature for 1 h. Later, the membranes were exposed dropwise to enterochromaffin‐like (ECL) luminescent reagent, and the grey values of each band were assessed by implementing Quantity ONE software [16].

### 2.8 Statistics

Data analysis and graphing were performed using GraphPad Prism 8.0 software (GraphPad Software Inc., San Diego, CA, USA). Quantitative data are presented as mean ± standard deviation (mean ± SD). Before data analysis, the Shapiro–Wilk test was used to assess data normality, and the Levene test was used to assess homogeneity of variance. For data that met the assumptions of normality and homogeneity of variance, independent sample t‐tests were used for comparisons between two groups, and one‐way analysis of variance (ANOVA) or two‐way ANOVA was used for comparisons among multiple groups. The factors analysed in two‐way ANOVA included (1) experimental treatment groups and (2) time points, with interaction effects considered. Post hoc tests for ANOVA were conducted using Tukey's post hoc test for data with homogeneous variance; if the data did not meet the assumption of homogeneity of variance, the Games‐Howell test was used instead. For analyses involving multiple comparisons, the Bonferroni correction method was used to adjust *p*‐values to reduce the false positive rate. A *p*‐value of < 0.05 was considered statistically significant.

## 3 Results

### 3.1 Effects of Different Doses of GS‐HCl on the Levels of Joint Injury Markers in KOA Mice

**FIGURE 1:**
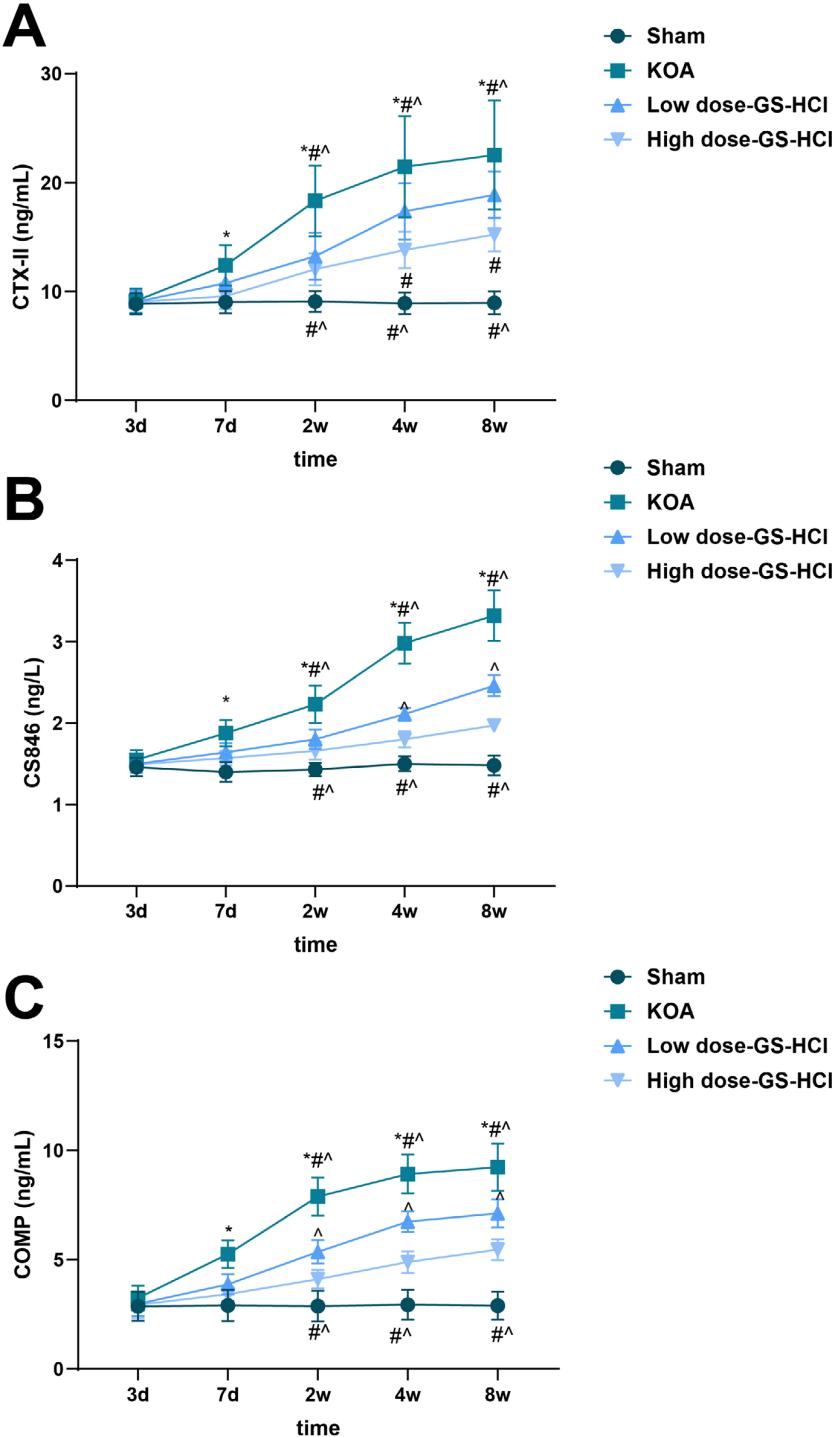
GS‐HCl treatment ameliorates joint injury in KOA mice. KOA mouse models were established by the ACLT method. (A–C) At 3 and 7 days as well as 2, 4 and 8 weeks after modelling, blood specimens were collected from each group of mice. Serum CTX‐II, CS846 and COMP levels were measured by ELISA. *n* = 6; Data are presented as mean ± standard deviation, and two‐way ANOVA was used for statistical analysis, followed by Tukey's post hoc test; *p < 0.05 vs. the Sham group, # *p* < 0.05 vs. the low‐dose GS‐HCl group and ^ *p* < 0.05 vs. the high‐dose GS‐HCl group.

The serum CTX‐II, CS846 and COMP levels in the KOA group were elevated from the 7th day after modelling compared with those in the Sham group. For KOA mice treated with different doses of GS‐HCl, their serum CTX‐II, CS846 and COMP levels were raised at the 2nd, 4th and 8th week after ACLT compared with those in the Sham group, but their serum CTX‐II, CS846, and COMP contents were lower at the 2nd, 4th and 8th week after ACLT compared with those in the KOA group. In addition, different doses of GS‐HCl treatment possessed significant effects on the alteration of serum CTX‐II, CS846 and COMP levels in KOA mice, and starting from the 4th week, serum CTX‐II, CS846 and COMP levels in the high‐dose GS‐HCl group were lower compared with those in the low‐dose GS‐HCl group (*p* < 0.05, Figure 1A–C). The above results suggested that GS‐HCl treatment could improve joint injury in KOA mice, and the effect was best at a high dose.

### 3.2 Effect of Different Doses of GS‐HCl on Histopathological Changes in the Cartilage of KOA Mice

**FIGURE 2:**
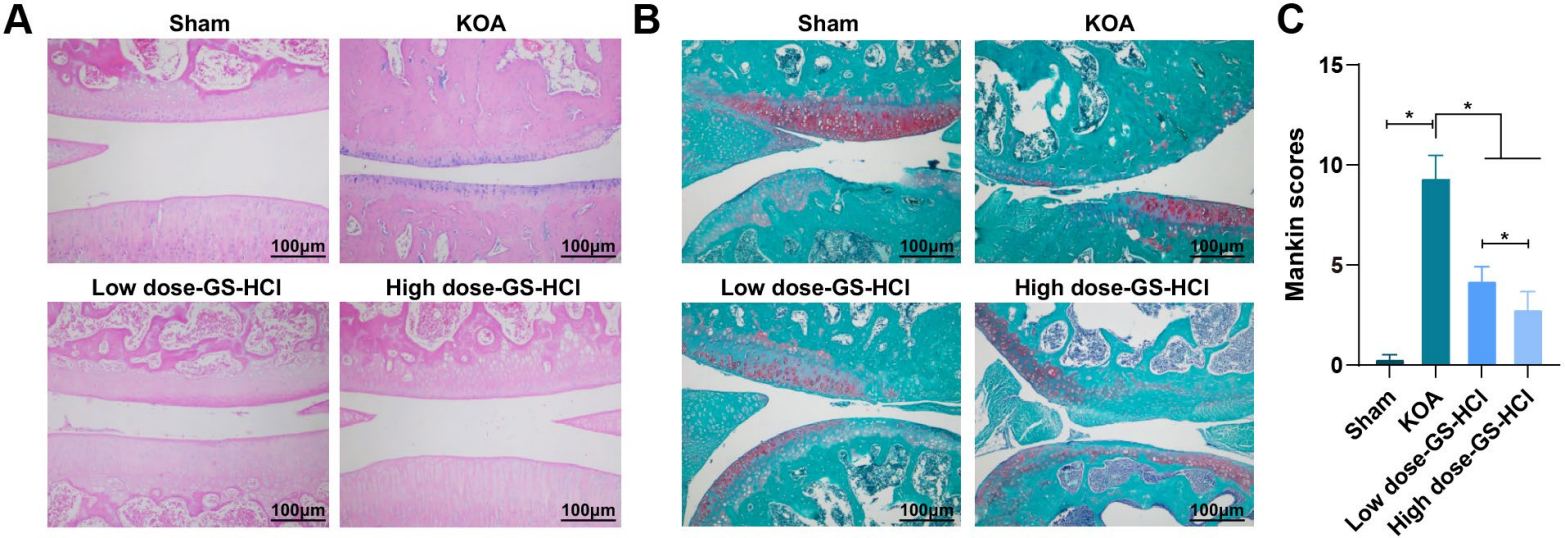
GS‐HCl reduces cartilage histopathological damage in KOA mice. (A) The histopathology of articular cartilage in KOA mice was observed by HE staining, with black arrows pointing to chondrocytes; (B) Safranin O staining was used to observe the proteoglycan content in the articular cartilage tissue of mice; (C) The degeneration of articular cartilage in knee femoral condyles of KOA mice was assessed by Mankin scores. *n* = 6; Data are presented as mean ± standard deviation, and one‐way ANOVA was used for statistical analysis, followed by Tukey's post hoc test; *p < 0.05.

We further observed the effects of different doses of GS‐HCl on the histopathological changes of cartilage in KOA mice. The findings of staining unravelled that in the Sham group, the cartilage surface was smooth; the chondrocytes were arranged in a regular manner and the tidemarks were clear. In the KOA group, the articular cartilage surface was irregular; the cartilage defect was severe; the articular cartilage surface was disordered at the defect; the chondrocytes had disappeared with the narrowing of the joint space, and there was a significant reduction in proteoglycans. Compared with the KOA group, the low‐dose GS‐HCl group exhibited a smoother cartilage surface, proliferation of chondrocytes, an increase in articular space and higher levels of proteoglycans. In the high‐dose GS‐HCl group, the articular surface was not only smoother but also flatter, with an increased number of chondrocytes, expanded articular space, elevated proteoglycan levels and an overall improved structural integrity (Figure 2A‐B). Tissue injury in each group of mice was scored according to microscopic performance at the meantime, and the results unearthed that the Mankin scores in the KOA group were higher compared with those in the Sham group. Different doses of GS‐HCl treatment were performed on KOA mice, which were able to reduce the degree of tissue injury in comparison with that in the KOA group, and the high‐dose GS‐HCl group possessed pronounced effects (*p* < 0.05, Figure 2C).

### 3.3 Effects of Different Doses of GS‐HCl on Apoptosis in KOA Mice

**FIGURE 3:**
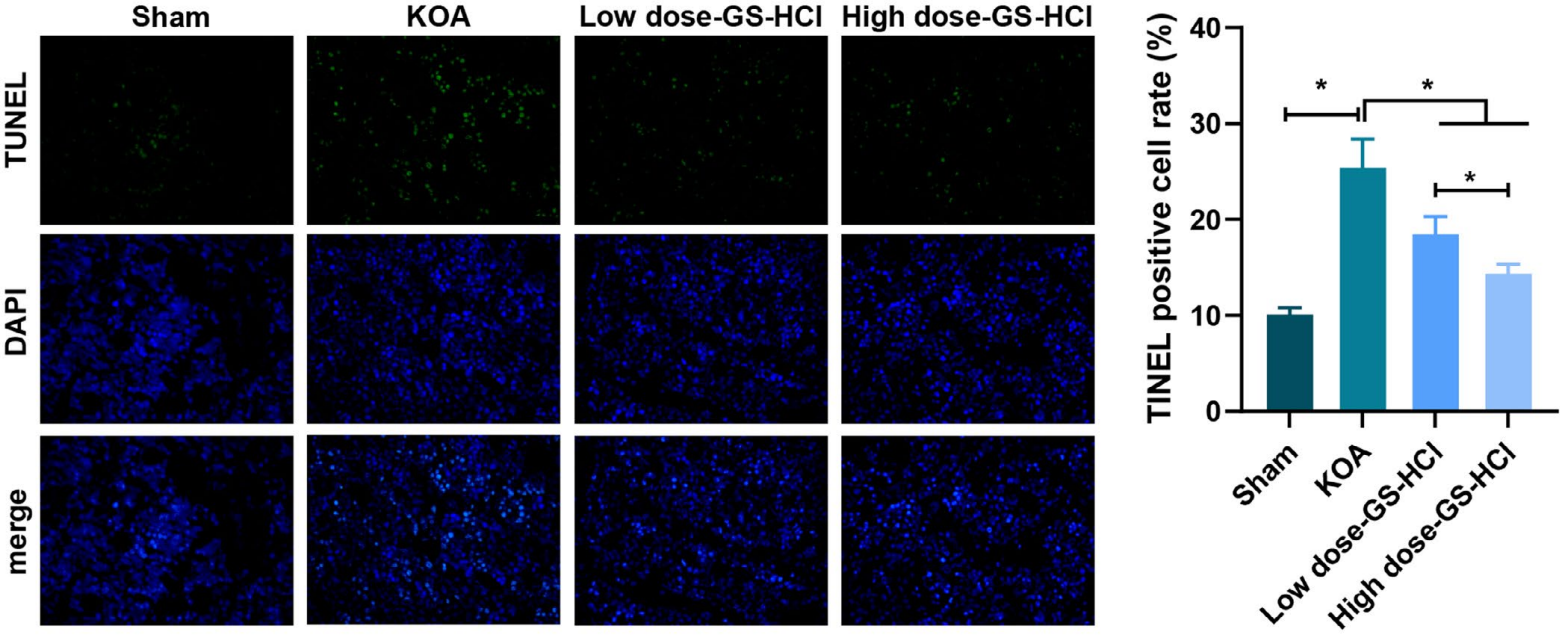
GS‐HCl diminishes apoptosis in cartilage tissue of KOA mice. TUNEL staining was utilised to test apoptosis in the tissues of KOA mice, *n* = 6; Data are presented as mean ± standard deviation, and one‐way ANOVA was used for statistical analysis, followed by Tukey's post hoc test; *p < 0.05.

To observe the impact of GS‐HCl on apoptosis in KOA mice, tissue sections from the knee joints of mice were taken for TUNEL staining, and the results unearthed that the apoptosis rate of the KOA group was higher compared with that of the Sham group; compared with the KOA group, KOA mice after treatment with different doses of GS‐HCl displayed reduced apoptosis in the tissues, of which the high dose GS‐HCl group possessed significant effects (*p* < 0.05, Figure 3).

### 3.4 Effects of Different Doses of GS‐HCl on Inflammation Response in KOA Mice

**FIGURE 4:**
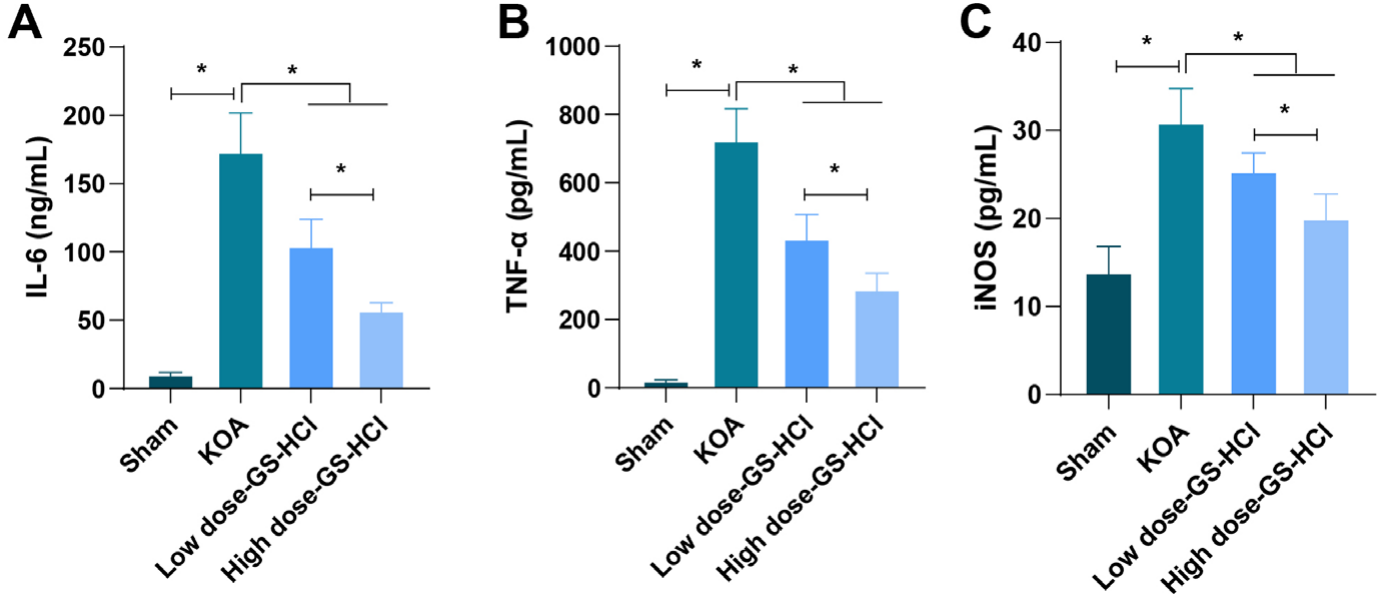
GS‐HCl treatment ameliorates inflammation responses in KOA mice. (A–C) The levels of IL‐6, TNF‐α and iNOS in the serum of KOA mice were tested by ELISA; *n* = 6; Data are presented as mean ± standard deviation, and one‐way ANOVA was used for statistical analysis, followed by Tukey's post hoc test; *p < 0.05.

Inflammatory responses were important pathological processes that promoted KOA development. This study measured the levels of inflammatory indicators in all groups of mice and found that IL‐6, TNF‐α and iNOS contents were higher in the KOA group in contrast with the Sham group, which confirmed that there were inflammation responses in KOA. Compared with the KOA group, KOA mice treated with low‐dose GS‐HCl and high‐dose GS‐HCl possessed diminished IL‐6, TNF‐α and iNOS contents, and the improvements in each indicator of the high‐dose GS‐HCl group were better (*p* < 0.05, Figure 4A–C).

### 3.5 Effects of Different Doses of GS‐HCL on Oxidative Stress Response in KOA Mice

**FIGURE 5:**
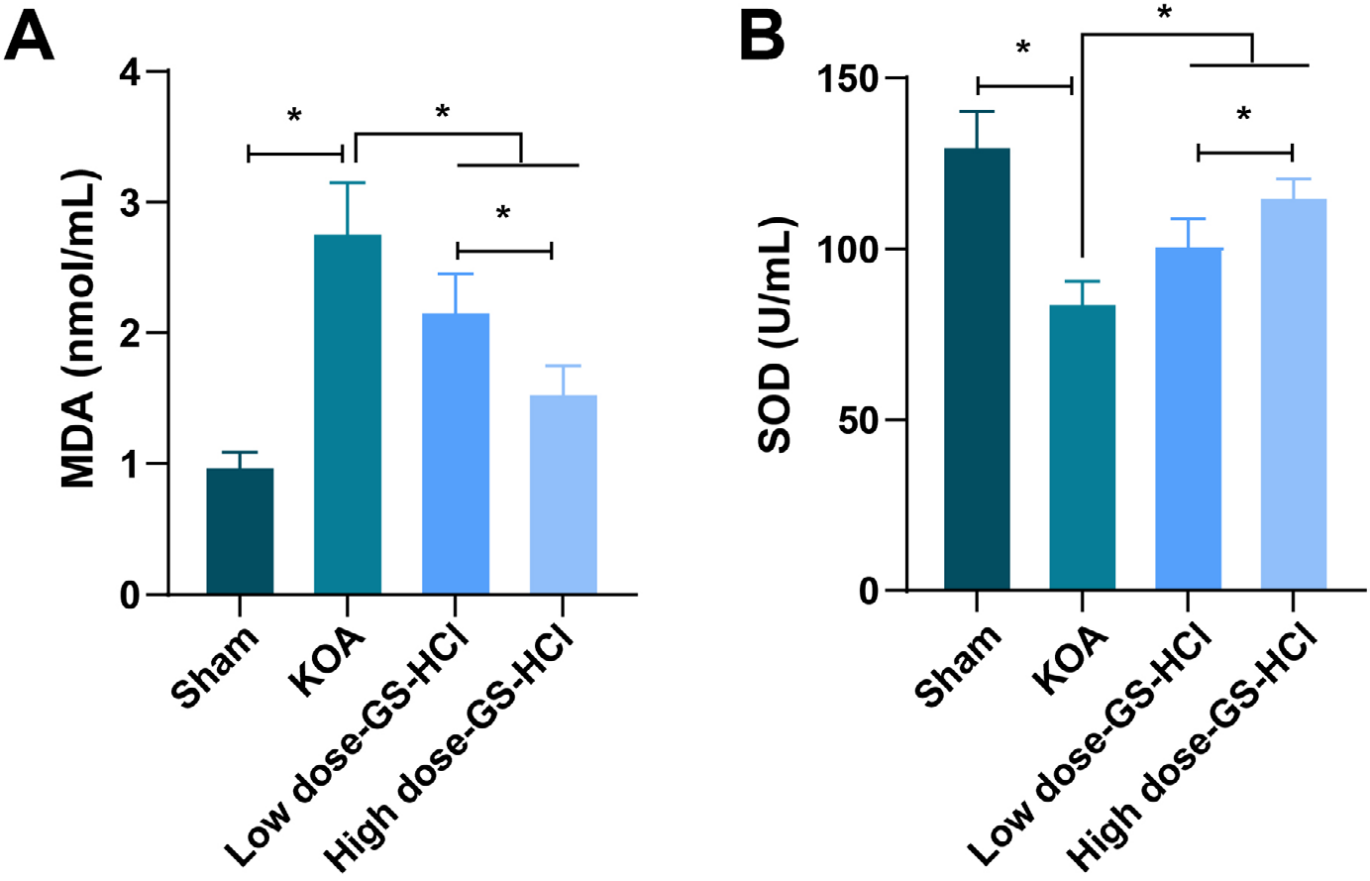
GS‐HCl treatment improves oxidative stress response in KOA mice. (A, B) MDA contents and SOD activity in the serum of KOA mice were assessed by kits; *n* = 6; Data are presented as mean ± standard deviation, and one‐way ANOVA was used for statistical analysis, followed by Tukey's post hoc test; *p < 0.05.

Further studies measuring the levels of oxidative stress indicators in each group of mice found that the MDA content in the KOA group was higher than that in the Sham group, while the SOD activity was lower than that in the Sham group (*p* < 0.05, Figure 5A,B). Compared with the KOA group, the MDA content in the low‐dose GS‐HCl group and the high‐dose GS‐HCl group was decreased, and the SOD activity was increased (*p* < 0.05, Figure 5A,B). Moreover, the improvement in each indicator in the high‐dose GS‐HCl group was better than that in the low‐dose GS‐HCl group (*p* < 0.05, Figure 5A,B).

### 3.6 Effects of Different Doses of GS‐HCl on Matrix Degradation in KOA Mice

**FIGURE 6:**
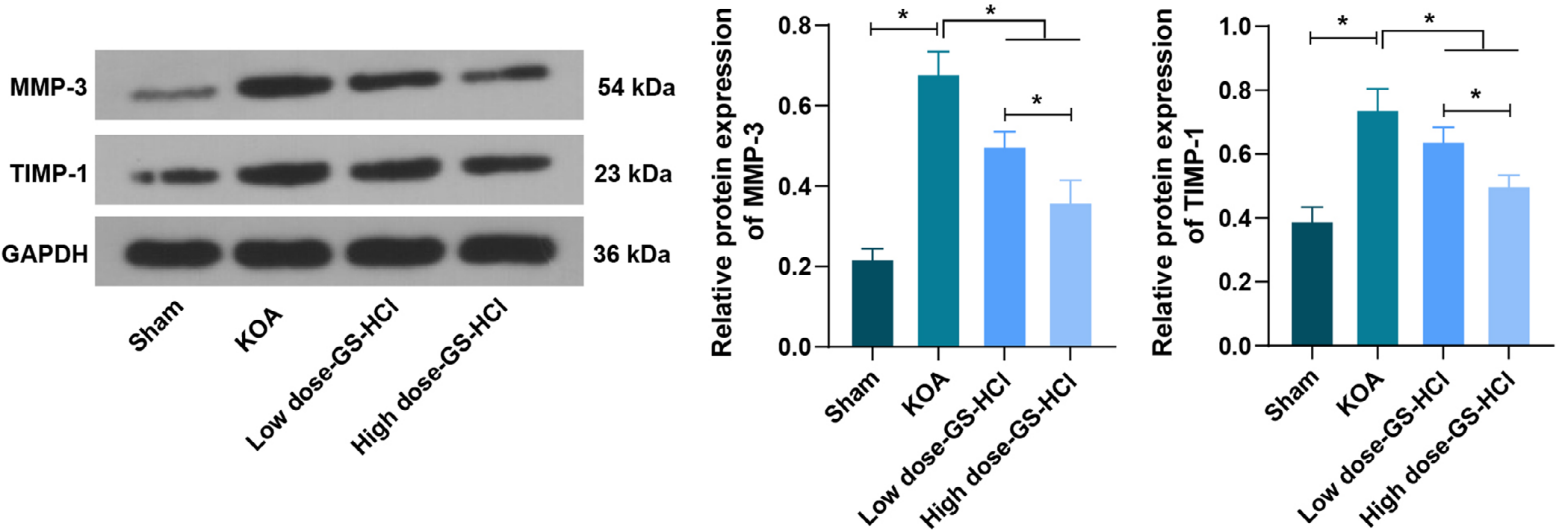
GS‐HCl treatment improves matrix degradation in KOA mice. MMP‐3 and TIMP‐1 protein expression in cartilage tissues of KOA mice after low‐dose GS‐HCl and high‐dose GS‐HCl treatment was tested by western blot assay. *n* = 6; Data are presented as mean ± standard deviation, and one‐way ANOVA was used for statistical analysis, followed by Tukey's post hoc test; *p < 0.05.

To observe the impact of GS‐HCl treatment on matrix degradation in KOA mice, MMP‐3 and TIMP‐1 expression in cartilage tissues was measured by western blot assay, and the findings unearthed that MMP‐3 and TIMP‐1 protein expression in cartilage tissues of the KOA group was up‐regulated compared with that of the Sham group, whereas GS‐HCl treatment was able to down‐regulate the MMP‐3 and TIMP‐1 protein contents in the cartilage of KOA mice. When comparing MMP‐3 and TIMP‐1 protein contents in cartilage tissues of KOA mice treated with low‐dose GS‐HCl and high‐dose GS‐HCl, it was revealed that the high‐dose GS‐HCl group achieved lower MMP‐3 and TIMP‐1 protein contents compared with the low‐dose GS‐HCl group (*p* < 0.05, Figure 6).

## 4 Discussion

KOA can lead to substantial pain and disability [22] and a substantial impact on quality of life, which is difficult to manage in the clinical setting [23]. This paper analysed the protective effects of different doses of GS‐HCl on cartilage tissue in KOA and its effects on the levels of joint injury markers.

Our research results indicated that different doses of GS‐HCl treatment possessed significant effects on the alteration of serum CTX‐II, CS846 and COMP levels in KOA mice, suggesting that GS‐HCl treatment can improve joint damage in KOA mice, with a more pronounced effect at higher doses. As previously reported, CTX‐II and COMP concentrations are raised after high‐dose GS‐HCl and low‐dose GS‐HCl treatment at 3 days after modelling [24]. Furthermore, in terms of cartilage tissue morphology, we revealed that GS‐HCl has a protective effect on cartilage tissue, promoting cartilage repair and maintaining normal structure, with better effects at higher doses. A previous study reports that GS‐HCl can exert a certain protective effect on cartilage after blood‐induced joint damage by downregulating IL‐1β, TNF‐α and MMP‐13, and increasing the content of proteoglycans in cartilage [24]. In view of this, compared with other studies, we emphasise the impact of different doses of GS‐HCl on the levels of joint damage markers throughout the treatment cycle. As the treatment progresses, especially from the 4th week onwards, the serum levels of CTX‐II, CS846 and COMP in the high‐dose GS‐HCl group are significantly lower than those in the low‐dose GS‐HCl group, further confirming the advantage of high‐dose GS‐HCl in improving joint damage. In addition, we have clarified the differences in improving cartilage tissue morphology between different doses of GS‐HCl, emphasising the significant advantage of high‐dose GS‐HCl in promoting cartilage repair and maintaining normal structure.

In a previous study, it is revealed that glucosamine sulfate can not only elevate type II collagen in human articular chondrocytes but also reduce cell death progression [25]. In our article, different doses of GS‐HCl treatment could significantly reduce tissue cell apoptosis, with a more significant effect in the high‐dose group. This indicates that GS‐HCl can inhibit chondrocyte apoptosis, helping to maintain the number and function of chondrocytes, and the inhibitory effect is stronger at higher doses.

The utilisation of GS‐HCl in KOA patients can achieve improved contents of inflammatory factors and promote immune function, which is manifested by lower TNF‐α and IL‐6 levels after GS‐HCl treatment [5]. Feng Wang et al. report that combining GS‐HCl with non‐steroidal anti‐inflammatory drugs can reduce the levels of inflammatory cytokines, alleviate knee pain and arthritis‐related symptoms and enhance knee function [11]. It has also been reported that GS‐HCl can prevent cartilage degradation mediated by the aggrecanases ADAMTS‐4 and ‐5 and reduce inflammatory responses. This may be one of the mechanisms by which GS‐HCl effectively maintains joint integrity and function, preventing or delaying the early symptoms of osteoarthritis [26]. Our research results have similarities with the abovementioned literature reports while also providing more detailed dose‐effect information. Our results confirmed that compared with the KOA group, the levels of IL‐6, TNF‐α and iNOS in both the low‐dose and high‐dose GS‐HCl groups were decreased, and the improvement in various indicators was better in the high‐dose group, suggesting that GS‐HCl can reduce the inflammatory response in KOA, with a better anti‐inflammatory effect at higher doses. Mechanistically, IL‐6 and TNF‐α are important pro‐inflammatory cytokines that can activate multiple inflammatory signalling pathways [27], leading to chondrocyte apoptosis, matrix degradation and joint inflammation. iNOS can induce the production of a large amount of nitric oxide, which has cytotoxic effects [28] and can aggravate cartilage damage. GS‐HCl may reduce the inflammatory response by inhibiting the production of these inflammatory factors, thereby reducing the damage of inflammation to chondrocytes and protecting cartilage integrity.

Khadijeh et al.'s study shows that GS‐HCl has dose‐dependent DPPH antioxidant activity and can effectively protect red blood cells from free radical damage, making it a recommended drug supplement for alleviating oxidative stress [29]. Their study suggests that GS‐HCl performs well in in vitro antioxidant activity, and its dose‐dependent characteristics indicate that the antioxidant effect may be further enhanced with increasing doses. Our study focuses on the impact of GS‐HCl on oxidative stress levels in a KOA model. Our study confirmed that compared with the KOA group, the MDA content in both the low‐dose and high‐dose GS‐HCl groups was decreased, and the SOD activity was increased, with a more significant improvement in the high‐dose group. This indicates that GS‐HCl can regulate oxidative stress levels, and reduce oxidative damage, and the regulatory effect is more obvious at higher doses. Mechanistically, MDA is a product of lipid peroxidation, and an increase in its content indicates an increase in oxidative stress levels [30]. SOD is an important antioxidant enzyme that can scavenge excess superoxide anion radicals in the body, reducing oxidative damage [31]. GS‐HCl may reduce oxidative damage and protect the structure and function of chondrocytes by regulating oxidative stress‐related indicators, thereby maintaining cartilage integrity.

In addition, GS‐HCl has a lasting effect on pain relief and functional improvement in KOA, exerting therapeutic effects by exerting anti‐inflammatory functions, inhibiting MMP activity, aminosugar degradation and nitric oxide production [32]. It is also observed the lower expression levels of relevant inflammatory factors and MMP after GS‐HCl treatment are also observed in previous research [11]. GS‐HCl is proven to have certain protective effects on cartilage via the downregulation of TNF‐α and MMP‐13. TNF‐α concentration after different doses of GS‐HCl treatment is higher at 8 weeks, and MMP‐13 expression is elevated [24]. Our study displayed that MMP‐3 and TIMP‐1 protein expression in the cartilage tissues of KOA mice was up‐regulated, whereas GS‐HCl treatment was able to down‐regulate the MMP‐3 and TIMP‐1 protein contents in cartilage of KOA mice. Moreover, high‐dose GS‐HCl achieved lower MMP‐3 and TIMP‐1 protein contents compared with low‐dose GS‐HCl. This indicates that GS‐HCl can affect the metabolism of the extracellular matrix and regulate the balance between MMP‐3 and TIMP‐1, with a more prominent regulatory effect at higher doses. Somewhat different from our research results, a study reveals that glucosamine methyl ester can prevent the degeneration of cartilage in rats with osteoarthritis. It exerts its effects by promoting collagen, type II collagen, albumin polysaccharides, TIMP‐1 and reducing MMP [33]. In view of this, glucosamine methyl ester focuses on inhibiting MMP activity by promoting the expression of TIMP‐1, while GS‐HCl may regulate the degradation and synthesis of the extracellular matrix in a more balanced way by downregulating MMP‐3 and TIMP‐1. Mechanistically, MMPs are a class of proteases that can degrade the extracellular matrix (ECM). Among them, MMP‐3 can degrade various ECM components, such as collagen and proteoglycans, leading to cartilage matrix degradation. TIMPs are natural inhibitors of MMPs and can inhibit MMP activity, protecting the ECM [34, 35]. GS‐HCl may reduce ECM degradation and promote the synthesis and repair of cartilage matrix by regulating the balance between MMP‐3 and TIMP‐1, thereby maintaining the structure and function of cartilage. It is worth noting that under certain pathological conditions, such as KOA, the overexpression of TIMP‐1 may be closely related to the inflammatory response and may even have an adverse effect on cartilage health. Therefore, GS‐HCl may reduce the inflammatory response in cartilage tissue by decreasing TIMP‐1 levels during the KOA process, thereby slowing down the progression of KOA.

In summary, this research demonstrates that the intragastric administration of the GS‐HCl group could partially prevent the degeneration of articular cartilage in KOA mice. The mechanism may be to diminish inflammatory factors and oxidative stress indicator expression and matrix degradation, thereby delaying osteoarthritis progress. This study lays a foundation to explore the protection of different doses of GS‐HCl for KOA and provides a reference for dose selection in clinical applications, helping to optimise treatment plans. Based on the results of this study, GS‐HCl is expected to become an effective therapeutic drug for KOA. In clinical practice, an appropriate dose can be selected for treatment based on the specific conditions of the patient (such as the severity of the disease, age and physical condition). GS‐HCl can be used as a monotherapy or in combination with other drugs (such as non‐steroidal anti‐inflammatory drugs and analgesics) to enhance the therapeutic effect. However, this study still has some limitations, such as the differences between the KOA mouse model and human OA pathology. Although the KOA mouse model can simulate certain pathological features of human KOA, there are still differences in the mechanisms of disease occurrence, development and clinical manifestations. Therefore, caution is needed when directly extrapolating the research results from the mouse model to humans. In addition, the long‐term efficacy and safety of GS‐HCl still require further research and verification. More data support is needed, especially regarding the effects of GS‐HCl on liver and kidney function and the potential risks of long‐term use. More clinical trials and long‐term follow‐up studies need to be conducted in the future to further verify the efficacy and safety of GS‐HCl and optimise treatment plans.

## Author Contributions

**Xichun Wang:** conceptualization (supporting), project administration (supporting), writing – review and editing (equal). **Bin Hu:** conceptualization (supporting), project administration (supporting), writing – review and editing (equal). **Yi Cheng:** conceptualization (supporting), project administration (supporting), writing – review and editing (equal). **Wenjie Chen:** conceptualization (supporting), project administration (supporting), writing – review and editing (equal). **Muzi Liu:** conceptualization (lead), project administration (lead), writing – original draft (lead).

## Ethics Statement

The experimental animals were utilised for medical research and all operations were conducted under discussion and ratification of the Animal Committee of Jiujiang City Key Laboratory of Cell Therapy, Jiujiang No. 1 People's Hospital.

## Consent

The authors have nothing to report.

## Conflicts of Interest

The authors declare no conflicts of interest.

## Data Availability

The data that support the findings of this study are available from the corresponding author upon reasonable request.
